# NOTCH-1 and NOTCH-4 are novel gene targets of PEA3 in breast cancer: novel therapeutic implications

**DOI:** 10.1186/bcr2900

**Published:** 2011-06-14

**Authors:** Anthony G Clementz, Allison Rogowski, Kinnari Pandya, Lucio Miele, Clodia Osipo

**Affiliations:** 1 Molecular and Cellular Biochemistry Program, Loyola University Medical Center, Maywood, IL 60153, USA; 2 Molecular Biology Program, Loyola University Medical Center, 2160 S. First Ave, Maywood, IL 60153, USA; 3 Cancer Institute of the University of Mississippi Medical Center, 2500 N. State St, Jackson, MS 39216, USA; 4 Oncology Institute, Loyola University Medical Center, 2160 S. First Ave, Maywood, IL 60153, USA; 5 Department of Pathology, Loyola University Medical Center, 2160 S. First Ave, Maywood, IL 60153, USA; 6 Department of Microbiology and Immunology, Loyola University Medical Center, 2160 S. First Ave, Maywood, IL 60153, USA

## Abstract

**Introduction:**

Women with triple-negative breast cancer have the worst prognosis, frequently present with metastatic tumors and have few targeted therapy options. *Notch-1 *and *Notch-4 *are potent breast oncogenes that are overexpressed in triple-negative and other subtypes of breast cancer. PEA3, an ETS transcription factor, is also overexpressed in triple-negative and other breast cancer subtypes. We investigated whether PEA3 could be the critical transcriptional activator of Notch receptors in MDA-MB-231 and other breast cancer cells.

**Methods:**

Real-time PCR and Western blot analysis were performed to detect *Notch-1*, *Notch-2*, *Notch-3 *and *Notch-4 *receptor expression in breast cancer cells when PEA3 was knocked down by siRNA. Chromatin immunoprecipitation was performed to identify promoter regions for *Notch *genes that recruited PEA3. TAM-67 and c-Jun siRNA were used to identify that c-Jun was necessary for PEA3 enrichment on the Notch-4 promoter. A Notch-4 luciferase reporter was used to confirm that endogenous PEA3 or AP-1 activated the *Notch-4 *promoter region. Cell cycle analysis, trypan blue exclusion, annexin V flow cytometry, colony formation assay and an *in vivo *xenograft study were performed to determine the biological significance of targeting PEA3 via siRNA, Notch signaling via a γ-secretase inhibitor, or both.

**Results:**

Herein we provide new evidence for transcriptional regulation of Notch by PEA3 in breast cancer. PEA3 activates *Notch-1 *transcription in MCF-7, MDA-MB-231 and SKBr3 breast cancer cells. PEA3 activates *Notch-4 *transcription in MDA-MB-231 cells where PEA3 levels are endogenously high. In SKBr3 and BT474 breast cancer cells where PEA3 levels are low, overexpression of PEA3 increases *Notch-4 *transcripts. Chromatin immunoprecipitation confirmed the enrichment of PEA3 on *Notch-1 *and *Notch-4 *promoters in MDA-MB-231 cells. PEA3 recruitment to *Notch-1 *was AP-1-independent, whereas PEA3 recruitment to *Notch-4 *was c-JUN-dependent. Importantly, the combined inhibition of Notch signaling via a γ-secretase inhibitor (MRK-003 GSI) and knockdown of PEA3 arrested growth in the G_1 _phase, decreased both anchorage-dependent and anchorage-independent growth and significantly increased apoptotic cells *in vitro*. Moreover, either PEA3 knockdown or MRK-003 GSI treatment significantly reduced tumor growth of MDA-MB-231 xenografts *in vivo*.

**Conclusions:**

Taken together, the results from this study demonstrate for the first time that *Notch-1 *and *Notch-4 *are novel transcriptional targets of PEA3 in breast cancer cells. Targeting of PEA3 and/or Notch pathways might provide a new therapeutic strategy for triple-negative and possibly other breast cancer subtypes.

## Introduction

Breast cancer continues to be the second leading cause of cancer-related deaths among women worldwide. Approximately 70% of breast cancers are estrogen receptor α-positive (ERα^+^) and progesterone receptor-positive (PR^+^). They are divided into two subtypes: luminal A, comprising those that are negative for the overexpression or gene amplification of *ErbB-2/HER2 *and have low levels of genes responsible for proliferation, and luminal B, comprising those that are positive for *HER2 *and have high expression of proliferation-associated genes [1,2]. This division is in part due to their sensitivity to antihormonal therapy such as tamoxifen or an aromatase inhibitor. The luminal A subtype carries the best prognosis, followed by luminal B. The third subtype is ER^-^/PR^- ^and HER2^+^, which contains gene amplification for the *ErbB-2/HER2 *oncogene. The HER2^+ ^subtype represents 15% to 25% of breast cancers and is currently treated with trastuzumab plus a taxane-based chemotherapy. The HER2^+ ^subtype of breast cancer is associated with excellent survival outcomes due to adjuvant trastuzumab therapy, a humanized monoclonal antibody that targets the HER2 receptor [3]. However, 30% to 60% of metastatic HER2^+ ^breast cancer are resistant to trastuzumab. The fourth subtype of breast cancer is the normal-like subtype, which resembles normal mammary epithelial cells expressing genes associated with adipose tissue. The fifth subtype represents 15% of breast cancers and is triple-negative, and thus lacks expression of ER/PR and HER2. Triple-negative breast cancers carry the worst prognosis because of the lack of US Food and Drug Administration-approved targeted therapies [4,5]. Thus, there is an immediate need for the elucidation of novel targets to treat women with triple-negative breast cancer and to increase these patients' overall survival.

Notch signaling has emerged as a target for the treatment of breast cancer [6]. In the mammalian system, there are four Notch receptors (Notch-1, Notch-2, Notch-3 and Notch-4) [7] and five known ligands (Delta-like 1, Delta-like 3 and Delta-like 4 and Jagged-1 and Jagged-2) [8-10]. Cell-to-cell contact is critical for the activation of Notch signaling, which subsequently enables the pathway to modulate genes involved in cell fate such as proliferation or differentiation [11]. Notch is processed in the trans-Golgi apparatus, where it undergoes the first of three proteolytic cleavages. The single polypeptide is cleaved (S1) by furin-like convertase forming the mature Notch receptor, which is a heterodimer consisting of Notch extracellular (NEC) and Notch transmembrane (NTM). The receptor is trafficked to the plasma membrane, where it awaits engagement with its membrane-associated ligand. Upon ligand-receptor engagement, the second cleavage (S2) by a disintegrin and metalloproteases 10 and 17 (ADAM10 and ADAM17, respectively) [12] releases NEC to be endocytosed into the ligand-expressing cell. Subsequently, NTM is cleaved (S3) by the γ-secretase complex, liberating the intracellular portion of Notch (NIC) [13]. NIC translocates to the nucleus and binds to CBF-1, a constitutive transcriptional repressor, displacing corepressors and recruiting coactivators such as Mastermind [14,15]. Notch activates many genes associated with differentiation and/or survival, including, but not limited to, the HES and HEY family of basic helix-loop-helix transcription factors [16], cyclin D1 [17] and c-Myc [18]. The third and final cleavage step is critical for active Notch signaling. Its inhibition can be exploited through emerging pharmacological drugs identified as γ-secretase inhibitors (GSIs), which attenuate signaling from all four receptors. Recent studies have demonstrated that GSI treatment suppresses breast tumor growth in a variety of breast cancer subtypes [19-23], providing evidence of novel therapeutic approaches.

The first evidence that Notch receptors are breast oncogenes was provided by mouse studies. Overexpression of constitutive, active forms of Notch-1 (N1IC) or Notch-4 (N4IC) form spontaneous murine mammary tumors *in vivo *[11]. Furthermore, elevated expression of Notch-1 and/or its ligand Jagged-1 in human breast tumors is associated with the poorest overall patient survival [24-26]. Recently, Notch-4 has been shown to be critical for the survival of tumor-initiating cells [27]. Similarly to studies performed using Notch-1, mouse mammary tumor virus (MMTV)-driven Notch-3 receptor intracellular domain expression in transgenic mice showed enhanced mammary tumorigenesis [28]. In HER2^- ^breast cancers, downregulation of Notch-3 resulted in suppressed proliferation and increased apoptosis [29]. In contrast, overexpression of Notch-2 in MDA-MB-231 cells significantly decreased tumor growth and increased apoptosis in vivo [30], suggesting that Notch-2 is a breast tumor suppressor.

The factors that regulate Notch receptor expression in breast cancer cells are still widely unknown. It has been shown that p53 binds to the Notch-1 promoter and activates Notch-1 receptor transcription in human keratinocytes [31]. Activator protein 1 (AP-1) has been demonstrated to be a transcriptional activator of Notch-4 in human vascular endothelial cells [32]. We asked which factors regulate Notch receptor transcription in breast cancer.

Polyomavirus enhancer activator 3 (PEA3/E1AF/ETV4) is a member of the ETS family of transcription factors, which also includes ERM and ER-81. PEA3 is overexpressed in metastatic breast carcinomas, particularly triple-negative breast tumors [33]. PEA3 regulates critical genes involved in inflammation and invasion, such as IL-8 [34], cyclooxygenase-2 (COX-2) [35] and matrix metalloproteases (MMPs) [36-39]. A dominant-negative form of PEA3 reduced tumor onset and growth in a MMTV/*neu*-transgenic model of breast cancer *in vivo *[40]. PEA3 contains an ETS winged helix-turn-helix DNA binding motif [41] that binds to the canonical sequence GGAA/T on target genes [42]. The affinity of binding relies on proximal sequences surrounding the ETS binding site which aid in transcriptional control based on context [43]. Phosphorylation of serine and threonine residues by the mitogen-activating protein kinase cascade activates PEA3 and is negatively regulated by the ubiquitin-proteasome pathway as well as by sumoylation [44-46].

The transcriptional activity of PEA3 is dependent on other activators to regulate gene transcription and is commonly partnered with AP-1 to regulate genes such as MMP-1, MMP-3, MMP-7 and MMP-9 [36]; urokinase-type plasminogen activator (uPA) [47]; COX-2 [35]; and ErbB-2 [48]. AP-1 is a dimeric complex consisting of the Fos (c-FOS, FosB, Fra-1 and Fra-2) and Jun (c-JUN, JunB and JunD) families [49]. Depending on the cellular context, AP-1 cooperates with other proteins including, but not limited to, NFκB, CBP/p300, Rb and PEA3 [50,51]. The functional role of AP-1 is to recruit and direct appropriate factors to regulate gene expression and promote proliferation, differentiation, inflammation and/or apoptosis [52].

Previous investigations have determined that overexpression of Notch-1 and Notch-4 plays a critical role in breast tumorigenesis [11] and that PEA3 overexpression is associated with aggressive breast cancers, particularly the triple-negative subtype [53-58]. Herein we provide novel evidence of a link between two pathways that are overexpressed in breast cancer. PEA3 is a transcriptional activator of Notch-1 and Notch-4 and a repressor of Notch-2 in MDA-MB-231 cells, an example of triple-negative breast cancer cells. PEA3-mediated Notch-1 transcription is AP-1-independent, while Notch-4 transcription requires both PEA3 and c-JUN. PEA3 and/or Notch signaling are essential for proliferation, survival and tumor growth of MDA-MB-231 cells. Furthermore, PEA3 is a transcriptional activator of both Notch-1 and Notch-4 in other breast cancer cells. Thus we hypothesized that targeting of the PEA3 and/or Notch pathways might provide a new therapeutic strategy for triple-negative breast cancer as well as possibly other breast cancer subtypes where PEA3 regulates Notch-1 and/or Notch-4.

## Materials and methods

### Cell culture and reagents

MDA-MB-231, SKBr3, BT474 and MCF-7 breast cancer cells were purchased from the American Type Culture Collection (Manassas, VA, USA). All cell lines were supplemented with 100 μmol nonessential amino acids and 1% L-glutamine. SKBr3 cells (supplemented with 10% fetal bovine serum (FBS)) and MDA-MB-231 cells (supplemented with 5% FBS) were maintained in Iscove's Minimal Essential Medium (IMEM). MCF-7 cells (supplemented with 10% FBS) were maintained in DMEM/Ham's Nutrient Mixture F-12. BT474 cells (supplemented with 10% FBS) were maintained in DMEM. All cells were cultured in a 37°C incubator with 5% CO_2_. MRK-003 GSI was kindly provided by Merck Oncology International, Inc. (33 Avenue Louis Pasteur, Boston, MA, USA). MRK-003 GSI was dissolved in dimethylsulfoxide (DMSO) and stored at -80°C until use. Lactacystin (L6785) was purchased from Sigma-Aldrich (St Louis, MO, USA), dissolved in deionized water and stored at -20°C until use. The pcDNA3.1 expression vector and the PEA3-pcDNA3.1 expression vector were kindly provided by Dr Mein-Chie Hung (The University of Texas MD Anderson Cancer Center, Houston, TX, USA). The pHMB empty and pHMB-TAM-67 expression vectors were kindly provided by Dr Richard Schultz, Department of Immunology and Microbiology, (Loyola University Medical Center, Maywood, IL, USA).

### RNA interference and reagents

Control scrambled siRNA-a (sc-37007), Notch-1 siRNA (sc-36095), and a smart pool of three distinct PEA3 siRNA (PEA3ia, sc-36205) were purchased from Santa Cruz Biotechnology (Santa Cruz, CA, USA). An unrelated control siRNA and PEA3 siRNA (PEA3ib, catalog number 115237) were purchased from Ambion (Austin, TX, USA). A smart pool of four distinct c-Jun siRNA (sc-29223) were purchased from Santa Cruz Biotechnology (Santa Cruz, CA, USA). Transfection reagents used were Lipofectamine 2000 and Lipofectamine RNAiMAX, which were purchased from Invitrogen (Carlsbad, CA, USA), and FuGENE 6 was purchased from Roche Diagnostics Corporation (Indianapolis, IN, USA). Protocols were performed as described by the manufacturers.

### Antibodies

Notch-1 (antibody clone C-20), Notch-4 (antibody clone H-225), PEA3 [16] (product number sc-113), c-JUN (antibody clone G-4) were purchased from Santa Cruz Biotechnology. β-actin (antibody clone AC-15) was purchased from Sigma-Aldrich and used as the loading control

### Real-time RT-PCR

Total RNA was extracted from the cells using the RNeasy Mini Kit (Qiagen, Valencia, CA, USA) according to the manufacturer's protocol. Total cDNA was reverse-transcribed from the total RNA with random hexamers using the MultiScribe™ Reverse Transcriptase Kit (Applied Biosystems, Foster City, CA, USA) according to the manufacturer's protocol. Analysis of transcript relative fold copy number adjusted to hypoxanthine-guanine phosphoribosyl transferase (HPRT), an endogenous control, was carried out by quantitative real-time PCR using iTaq™ SYBR Green Supermix with ROX (Bio-Rad Laboratories, Hercules, CA, USA) and the PlusOne thermal cycler from Applied Biosystems. The following primers were used for detection: Notch-1 (forward primer: 5'-GTCAACGCCGTAGATGACC-3', reverse primer: 5'-TTGTTAGCCCCGTTCTTCAG-3'), Notch-2 (forward primer: 5'-TCCACTTCATACTCACAGTTGA-3', reverse primer: 5'-TGGTTCAGAGAAAACATACA-3'), Notch-3 (forward primer: 5'-GGGAAAAAGGCAATAGGC-3', reverse primer: 5'-GGAGGGAGAAGCCAAGTC-3'), Notch-4 (forward primer: 5'-AACTCCTCCCCAGGAATCTG-3', reverse primer: 5'-CCTCCATCCAGCAGAGGTT-3'), PEA3 (forward primer: 5'-AGGAGACGTGGCTCGCTGA-3'), (reverse primer: 5'-GGGGCTGTGGAAAGCTAGGTT-3'), HEY-1 (forward primer: 5'-TGGATCACCTGAAAATGCTG-3', reverse primer: 5'-TTGTTGAGATGCGAAACCAG-3'), MMP-9 (forward primer: 5'-TCGTGGTTCCAACTCGGTTT-3', reverse primer: 5'-GCGGCCCTCGAAGATGA-3') and IL-8 (forward primer: 5'-CACCGGAAGGAACCATCTCACT-3', reverse primer: 5'-TCAGCCCTCTTCAAAAACTTCTCC-3'), with HPRT (forward primer: 5'-ATGAACCAGGTTATGACCTTGAT-3', reverse primer: 5'-CCTGTTGACTGGTCATTACAATA-3') used as the loading control. Protocols were performed as described by the manufacturers. Transfection conditions for Western blot analysis and RT-PCR were as follows. MDA-MB-231 (4 × 10^5^), MCF-7 (4 × 10^5^), SKBr3 (5 × 10^5^) and BT474 (5 × 10^5^) cells were placed into a six-well culture dish. Twenty-four hours later the cells were transfected with reagents. Forty-eight hours later cells were harvested and protein/RNA was extracted. Notch-4 luciferase constructs were generously provided by Dr Emery Bresnick, Cell & Regenerative Biology, (University of Wisconsin at Madison, Madison, WI, USA). The AP-1 luciferase construct was generously provided by Dr Richard Schultz, Department of Immunology and Microbiology,(Loyola University Medical Center, Maywood, IL, USA). Dual-luciferase assays were performed as described by the manufacturer (Promega, Madison, WI, USA). A pRL-thymidine kinase promoter-driven Renilla luciferase reporter was cotransfected with the Firefly luciferase construct mentioned above as an internal transfection control. Transfection activity was measured using the Veritas Microplate Luminometer (Turner Biosystems, Sunnyvale, CA, USA) and represented as the ratio of Firefly luciferase to Renilla luciferase.

### Western blot analysis

The cells were lysed in radioimmunoprecipitation assay buffer (pH 8.0) containing 50 mmol Tris HCl, 150 mmol NaCl, 1% Nonidet P-40 (NP-40), 0.5% sodium deoxycholate, 0.1% SDS, 25 mmol β-glycerophosphate, 1 mmol sodium orthovanadate, 1 mmol sodium fluoride, 1 mmol phenylmethylsulfonyl fluoride, 1 mg/mL aprotinin and 1 mg/mL leupeptin. Western blot analysis was performed as previously described [21]. NuPAGE Bis-Tris Gels (4% to 12%; Invitrogen) in 3-(N-morpholino)propanesulfonic acid buffer were run at 175 V for 1.5 hours, and proteins were transferred at 38 V for 2 hours using polyvinylidene fluoride membranes. Protein detection was performed using the SuperSignal West Dura Substrate (Thermo Scientific, Rockford, IL, USA) and visualized by using the FUJIFILM™ Las-300 imager (GE-Healthcare Bio-Sciences, Piscataway, NJ, USA).

### Chromatin immunoprecipitation

MDA-MB-231 cells (3 × 10^6^) were plated in 150-cm^2 ^Petri dishes. Twenty-four hours later cells were transfected with pcDNA3.1, PEA3-pcDNA3.1 and PEA3-pcDNA3.1, together with PEA3 siRNA, for 48 hours. The cells were cross-linked with 1% formaldehyde and lysed in SDS lysis buffer (1% SDS, 10 mmol ethylenediaminetetraacetic acid (EDTA), 50 mmol Tris HCl, pH 8.1). The lysates were sonicated using the Branson Sonifier model 250 (Branson Ultrasound Corp., Danbury, CT, USA) at output 4.5, duty cycle 50, and pulsed 10 times. The lysate was then diluted 1:10 in immunoprecipitation dilution buffer (0.01% SDS, 1.1% Triton X-100, 1.2 mmol EDTA, 16.7 mmol Tris HCl, 167 mmol NaCl, pH 8.1). Approximately 300 to 700 μg of total precleared protein in lysates were incubated with 4 μg of PEA3 antibody (sc-113X; Santa Cruz Biotechnology) or mouse immunoglobulin G (IgG) overnight. Fifty microliters of protein G-plus agarose beads (sc-2002; Santa Cruz Biotechnology) were added to the immune complexes for 2 hours while gently rocking. Immune complexes/beads were washed in low-salt buffer (0.1% SDS, 1% Triton X-100, 2 mmol EDTA, 20 mmol Tris HCl, 150 mmol NaCl, pH 8.1), high-salt buffer (0.1% SDS, 1% Triton X-100, 2 mmol EDTA, 20 mmol Tris HCl, 500 mmol NaCl, pH 8.1), LiCl buffer (1% sodium deoxycholate, 1% NP-40, 0.25 mol LiCl, 1 mmol EDTA, 10 mmol Tris HCl, pH 8.1) and Tris-EDTA buffer (1 mmol EDTA and 10 mmol Tris HCl) at 4°C with agitation. The protein/antibody complexes from beads were eluted in freshly prepared elution buffer (1% SDS, 0.1 mol sodium bicarbonate, pH 8.0). Cross-linking of proteins and DNA was reversed by heating at 65°C overnight while gently rocking. The protein was degraded using a proteinase solution (0.5 mol EDTA, 1 M Tris HCl, pH 8.0, 10 mg/mL proteinase K) and incubated at 52°C for 1 hour. DNA was isolated using the QIAquick PCR purification kit (Qiagen). Enrichment at promoter sites was detected by quantitative PCR using iTaq™ SYBR Green Supermix with ROX (Bio-Rad Laboratories). *C*_t _threshold values were normalized to the IgG control. The following promoter region primers were used in detection: Notch-1 promoter regions not containing ETS or AP-1 sequences served as negative controls (forward primer: 5'-GTGCACACGGCTGTCCG-3', reverse primer: 5'-GCGACAACTGGCGACTGAA-3'), 2× PEA3 (forward primer: 5'-GCTGCAAGAGCCAAGATGAA-3', reverse primer: 5'-GGTGCCTGTGTTGAAAGCTCT-3'), c-ETS (forward primer: 5'-CTCCTGGCGCTTAACCAGG-3', reverse primer: 5'-CCAGAAAGCACAAACGGGTC-3'), AP-1(A) (forward primer: 5'-GCCTCCTTAGCTCACCCTGA-3'), reverse primer (5'-TCTTCAGAGGCCCCCTGC-3'), AP-1(B) (forward primer: 5'-TCCGCAAACCAGGCTCTG-3', reverse primer: 5'-ATTGGGGTGCAGTGCCG-3'), Sp-1/PEA3 (forward primer: 5'-CACCTCGACTCTGAGCCTCAC-3', reverse primer: 5'-CTCTTCCCCGGCTGGCT-3'). Notch-4 promoter regions not containing ETS or AP-1 sequences served as negative controls (forward primer: 5'-GGCGAGGATTCTAATGTGGA-3', reverse primer: 5'-CCCTGA GTGAAAGGGTGAAG-3'), CBF-1 (forward primer: 5'-TGGTACCACCCTGGTCAGTAT-3', reverse primer: 5'-TGCTCAGGCATTATGAGCTATG-3'), AP-1(A) (forward primer: 5'-AGCTGCCACTGACACCTTCT-3', reverse primer: 5'-CAGGGAATGCCAGTCAGAAT-3'), AP-1(B) (forward primer: 5'-ACTTCCCCAGGGGTTGTC-3', reverse primer: 5'-CTTCCTCCTCGGCCTGCT-3') and PEA3/ETS (forward primer: 5'-GGGTTCCTCTTCCCCATACC-3', reverse primer: 5'-TCATTTTCCCATCACCTTCCTT-3').

### Coimmunoprecipitation

MDA-MB-231 cells (3 × 10^6^) were plated in 150-cm^2 ^Petri dishes for 24 hours. The cells were transfected with pcDNA3.1, PEA3-pcDNA3.1 and PEA3-pcDNA3.1, together with PEA3 siRNA, for 48 hours. The cells were cross-linked with 1% formaldehyde and lysed in SDS lysis buffer (1% SDS, 10 mmol EDTA, 50 mmol Tris HCl, pH 8.1). The lysates were sonicated using the Branson Sonifier 250 at output 4.5, duty cycle 50, and pulsed 10 times. The lysate concentration was ascertained and was equally diluted in immunoprecipitation dilution buffer (0.01% SDS, 1.1% Triton X-100, 1.2 mmol EDTA, 16.7 mmol Tris HCl, 167 mmol NaCl, pH 8.1). Approximately 300 to 700 μg of total precleared lysates were incubated with 3 μg of PEA3 antibody (sc-113X; Santa Cruz Biotechnology) or mouse IgG overnight. Thirty microliters of protein G-plus agarose beads (sc-2002, Santa Cruz Biotechnology) were added to the immune complexes for 2 hours while gently rocking. Immune complexes and beads were washed three times in PBS. The pellet was resuspended in Laemmli sample buffer with 5% β-mercaptoethanol (Bio-Rad Laboratories) and heated for 5 minutes at 95°C while vigorously shaking. Western blot analysis was used to detect coimmunoprecipitation proteins as described above. Antibodies used for detection were PEA3 (16), c-FOS (H-125), c-JUN (G-4) and Fra-1 (R-20).

### Cell cycle analysis

MDA-MB-231 cells (1 × 10^5^) were seeded into a six-well plate. After 24 hours, the cells were treated/transfected with DMSO/scrambled, control siRNA, DMSO/PEA3 siRNA, MRK-003 (10 μmol)/scrambled siRNA or MRK-003 (10 μmol)/PEA3 siRNA. Briefly, 24 and 48 hours posttreatment/posttransfection, the cells and media were isolated and permeabilized with 70% ethanol. The cells were then pelleted, washed in 5% bovine calf serum and resuspended in 10 μg/mL RNase/PBS solution. The cells were stained with propidium iodide (100 μg/mL) and analyzed by flow cytometry (FACSCanto; BD Biosciences, San Jose, CA, USA).

### Annexin V

MDA-MB-231 cells (1.5 × 10^5^) were seeded into a six-well plate for 24 hours. Cells were treated/transfected with DMSO/scrambled siRNA, DMSO/PEA3 siRNA, MRK-003 (10 μmol)/scrambled siRNA and MRK-003 (10 μmol)/PEA3 siRNA. Briefly, after 24 and 48 hours of treatment/transfection, 1 × 10^6 ^cells were isolated in 1× annexin binding buffer (0.1 mol 4-(2-hydroxyethyl)-1-piperazineethanesulfonic acid/NaOH, 140 mol NaCl, 25 mmol CaCl_2_, pH 7.4) and treated with both fluorescein isothiocyanate-annexin V stain and propidium iodide. After 1-hour incubation, the samples were subjected to analysis by flow cytometry (FACSCanto; BD Biosciences).

### Cell viability assay

MDA-MB-231 cells (1.5 × 10^5^) were seeded into a six-well plate for 24 hours. Cells were treated/transfected with DMSO/scrambled siRNA, DMSO/PEA3 siRNA, MRK-003 (10 μmol)/scrambled siRNA and MRK-003 (10 μmol)/PEA3 siRNA. Briefly, after 24 and 48 hours of treatment/transfection, cells and media were harvested and washed in PBS. The cells were stained with trypan blue and counted under a standard slide microscope at ×40 magnification.

### Colony formation assay

In a six-well plate, a bottom layer of 1 mL of 0.8% agar dissolved in IMEM was added. Two milliliters of a 2% methylcellulose/IMEM solution (supplemented with 5% FBS, 1% nonessential amino acids and 1% L-glutamine) were added on top of the agar. MDA-MB-231 cells (1 × 10^3 ^cells/mL) were treated/transfected with DMSO/scrambled siRNA, DMSO/PEA3 siRNA, MRK-003 (5 μmol)/scrambled siRNA or MRK-003 (5 μmol)/PEA3 siRNA for 24 hours. Treated cells were added directly to the methylcellulose solution. The assay was left untouched for 14 days at 37°C and 5% CO_2_. Colonies were stained with crystal violet for 1 hour, photographed and counted under a standard light microscope at ×40 magnification. Nine fields per well were counted.

### MDA-MB-231 xenograft study

MDA-MB-231 cells were transfected *in vitro *with control or PEA3 siRNA smart pool for 24 hours as described previously. One million cells were subsequently injected into each of two mammary fat pads of 10 Balb/c athymic nude mice per group, for a total of 40 mice. The mice were randomized to control siRNA or PEA3 siRNA and fed orally by gavage, vehicle control (2% carboxymethylcellulose in sterile PBS) or 100 mg/kg MRK-003 GSI for three consecutive days per week. The tumor areas were measured using vernier calipers, and growth rates were calculated by linear regression analysis. The tumor areas were monitored biweekly for up to 3.5 weeks. The protocols used to study breast tumor xenografts in mice were approved by Loyola University's Institutional Animal Care and Use Committee.

## Results

### PEA3 regulates Notch-1, Notch-2 and Notch-4 receptor transcripts

**Figure 1:**
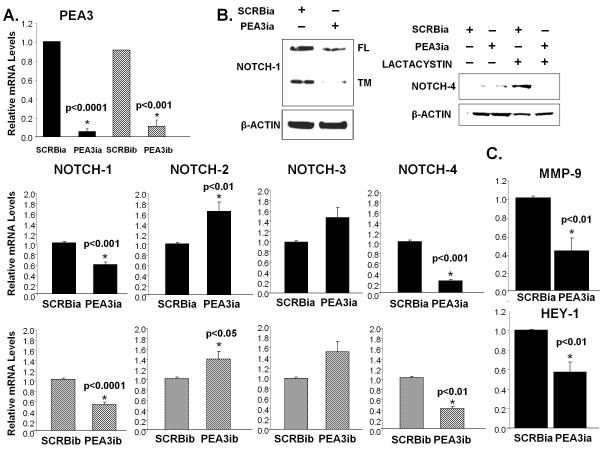
**PEA3 positively regulates Notch-1 and Notch-4 transcription**. MDA-MB-231 cells were transfected with one of two unrelated scrambled siRNA (SCRBia or SCRBib) or one of two unrelated PEA3 siRNA (PEA3ia or PEA3b). **(A) **Relative mRNA levels of Notch receptors (NOTCH-1, NOTCH-2, NOTCH-3 and NOTCH-4) were measured by quantitative PCR. Relative mRNA levels of PEA3 were measured as knocked-down controls using quantitative PCR. **(B) **Protein levels of Notch-1 and Notch-4 were detected by Western blot analysis. For Notch-4 Western blot analysis, cells were treated with or without lactacystin for 24 hours. The Western blots are representative of three independent experiments. **(C) **Downstream targets of PEA3 (MMP-9) and Notch (HEY-1) were measured by quantitative PCR. Transcripts were normalized to HPRT, and mean values were plotted. Error bars represent standard deviations. The statistical significance of three or more experiments was determined by performing a two-tailed, unpaired Student's *t*-test for two comparisons.

To investigate the role of PEA3 in *Notch *expression, we examined the effect of two independent PEA3 RNA interference sequences on the expression of *Notch-1 *through *Notch-4 *receptor mRNA as measured by real-time PCR. *Notch-1 *and *Notch-4 *transcripts were decreased by nearly 50% and 70%, respectively, upon PEA3 knockdown by either PEA3ia or PEA3ib siRNA. Notch-3 transcript levels remained unchanged. Interestingly, *Notch-2 *levels showed a moderate but significant increase upon PEA3 knockdown, which, upon further investigation, may prove to be advantageous, since it has been correlated with proapoptotic and breast tumor suppressive function [30] (Figure 1A, lower left graphs). PEA3 transcripts were measured as a control for the efficacy of PEA3 knockdown using the two types of PEA3 siRNA (Figure 1A, upper left graph).

To determine whether the effect on *Notch-1 *and *Notch-4 *transcripts correlated with protein expression, protein lysates from cells transfected with either a scrambled, control siRNA (SCRBia) or PEA3 siRNA (PEA3ia) for 48 hours were subjected to Western blot analysis. The results showed a reduction of Notch-1 full-length and transmembrane receptor protein levels by 50% (Figure 1B, upper right panel). To visualize the effects of PEA3 siRNAa on the rapidly turned over Notch-4 receptor, we treated MDA-MB-231 cells with or without lactacystin, a specific proteasome inhibitor, for 24 hours before lysing cells for total proteins. Consequently, N4IC protein levels were decreased by 90% upon PEA3 knockdown (Figure 1B, upper right panel).

To confirm the effect of PEA3 siRNAa on the levels and activity of PEA3, PEA3 and its classical downstream targets were measured by real-time PCR after transfection with control or PEA3 siRNA for 48 hours. PEA3 transcripts were significantly reduced (Figure 1A, left graph) as well as its known target gene *MMP-9 *(Figure 1C). Moreover, *HEY-1*, a classic downstream target of the Notch signaling pathway, was also significantly reduced upon PEA3 knockdown. Taken together, these results suggest that PEA3 positively regulates *Notch-1 *and *Notch-4 *receptor expression and affects Notch activity in MDA-MB-231 cells.

### *PEA3 regulates Notch-1 and Notch*-4 expression in other subtypes of breast cancer

**Figure 2:**
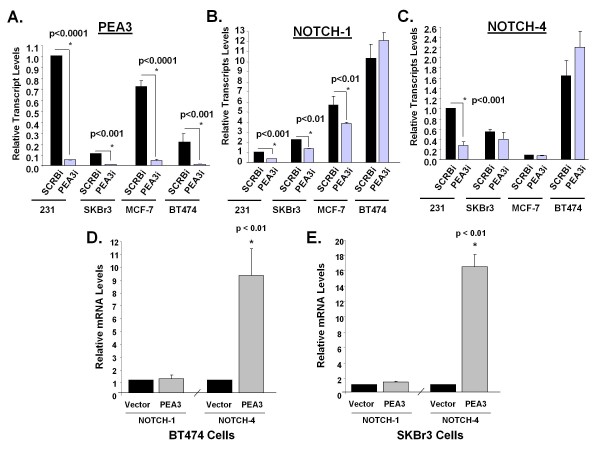
**PEA3 regulates Notch-1 and Notch-4 in different subtypes of breast cancer**. **(A) **through **(C) **MDA-MB-231, MCF-7, BT474 and SKBr3 cells were transfected with either scrambled or PEA3 siRNAa. Once normalized to MDA-MB-231 cells, the transcript levels of PEA3 **(A)**, Notch-1 **(A) **and Notch-4 **(C) **were compared and measured by quantitative real-time PCR. BT474 cells **(D) **and SKBr3 cells **(E) **were transfected with either empty vector or PEA3 expression plasmid. Notch-1 and Notch-4 mRNA levels were normalized to empty vector and measured by quantitative PCR. Means and standard deviations of three experiments were plotted. Statistical significance was determined by performing a two-tailed, unpaired Student's *t*-test.

Our investigations are the first to show that PEA3 is a transcriptional activator of *Notch-1 *and *Notch-4 *in MDA-MB-231 breast cancer cells, an example of triple-negative breast cancer. We next assessed whether this is a common mechanism among the other subtypes of breast cancer cells. We used the single pool of PEA3 siRNAa because similar results were obtained for the two independent siRNA. MDA-MB-231 (triple-negative), MCF-7 (luminal A), BT474 (luminal B) and SKBr3 (HER2^+^) cells were transfected with either scrambled or PEA3 siRNAa. Normalized to MDA-MB-231 cells, MCF-7 cells had slightly less PEA3 transcripts, followed by BT474 cells and then SKBr3 cells (Figure 2A). *Notch-1 *(Figure 2B) and *Notch-4 *(Figure 2C) transcript levels were compared with either scrambled or PEA3 siRNA among the cell lines. PEA3 regulation of *Notch-1 *was seen in MDA-MB-231, SKBr3 and MCF-7 cells, but not BT474 cells (Figure 2B). However, PEA3-mediated effects on *Notch-4 *transcripts were observed only in MDA-MB-231 cells (Figure 2C). This could be due in part to the fact that PEA3 basal transcript levels were very low in other cell types (Figure 2A). Motivated by this notion, we asked whether we could drive the expression of *Notch-4 *by increasing levels of PEA3 in breast cancer cells that express low endogenous PEA3 levels. To address this question, BT474 and SKBr3 cells were transfected with either pcDNA3.1 vector alone or PEA3-pcDNA3.1 expression plasmid. *Notch-1 *transcripts remained unchanged when PEA3 was overexpressed (Figures 2D and 2E). *Notch-4 *transcript levels were significantly increased ninefold in BT474 (Figure 2D) and sixteenfold in SKBr3 breast cancer cells (Figure 2E).

Taken together, PEA3 is required for *Notch-1 *transcription in at least three subtypes of breast cancer cells: MDA-MB-231, SKBr3 and MCF-7. However, PEA3 is an activator of *Notch-4 *transcription in MDA-MB-231 cells where endogenous PEA3 levels are high. Exogenous expression of PEA3 in BT474 and SKBr3 cells provides evidence that it is also a potential activator of *Notch-4 *transcription in other breast cancer subtypes.

### PEA3 is enriched on the Notch-1 and Notch-4 promoters

**Figure 3:**
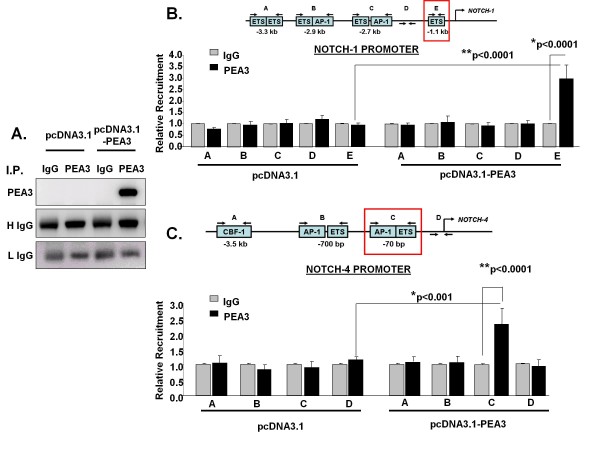
**PEA3 is enriched on the Notch-1 and Notch-4 promoters**. **(A) **MDA-MB-231 cells were transfected with pcDNA3.1 alone or pcDNA3.1-PEA3 expression plasmid. Lysates were immunoprecipitated (I.P.) with IgG control or PEA3-specific antibody. Western blot analysis was performed to detect PEA3. Heavy and light chains of IgG were used for loading. The Western blots are representative of three independent experiments. **(B) **and **(C) **MDA-MB-231 cells transfected with pcDNA3.1 or pcDNA3.1-PEA3 expression plasmid were lysed, and lysates were subjected to chromatin immunoprecipitation using either PEA3-specific antibody or an isotype control IgG on the Notch-1 promoter **(B) **or the Notch-4 promoter **(C)**, respectively. PEA3 enrichment was measured by quantitative PCR and normalized to pcDNA3.1 and IgG control. Schematic representations of Notch-1 and Notch-4 promoter regions and the primers used in the studies flanking the specific sites are shown. Error bars represent standard deviations. Statistical significance of three or more experiments was determined by performing a two-tailed, unpaired Student's *t*-test for two comparisons.

To determine the mechanism by which PEA3 regulates Notch-1 and Notch-4 transcription, we scanned the promoter regions of *Notch-1 *and *Notch-4 *using the National Center for Biotechnology Information Entrez GenBank [59,60] and identified several ETS binding sites (Figures 3B and 3C, respectively). Interestingly, both promoters contained two AP-1 sites adjacent to ETS sites. A classic CBF-1 site was also previously identified in the *Notch-4 *promoter [23] and was used as a negative control for PEA3 recruitment. Primers were subsequently designed to include these identified regions. Primers flanking promoter regions containing no known consensus sites were used as negative controls. We then performed chromatin immunoprecipitation (ChIP) experiments on lysates from MDA-MB-231 cells that were transfected with either pcDNA3.1 vector alone or pcDNA3.1-*PEA3 *expression plasmid.

**Figure 4:**
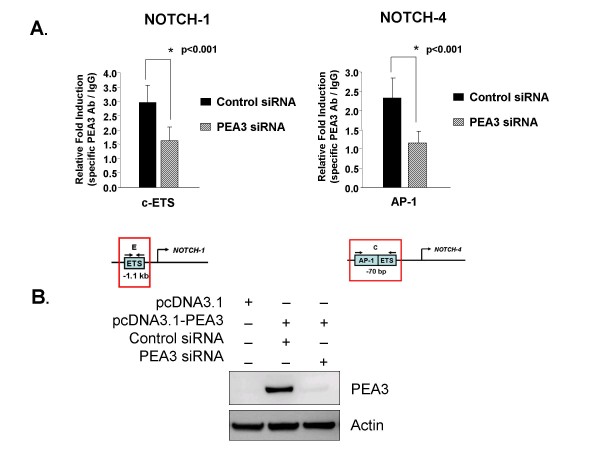
**PEA3 is required for enrichment in Notch-1 and Notch-4 promoter regions**. **(A) **MDA-MB-231 cells were cotransfected with pcDNA3.1-PEA3 expression plasmid and control siRNAa or PEA3 siRNAa. Lysates were subjected to chromatin immunoprecipitation using either PEA3-specific antibody or an isotype control IgG on the Notch-1 and Notch-4 promoters, respectively. PEA3 enrichment was measured by quantitative PCR and normalized to IgG control. Statistical comparisons were performed between PEA3 siRNAa and control siRNAa using a two-tailed, unpaired Student's *t*-test for two comparisons. **(B) **Lysates from the experiment described in **(A) **were subjected to SDS-PAGE followed by Western blot analyses to detect PEA3 protein and actin protein as a loading control. The Western blots are representative of three independent experiments. Error bars represent standard deviations. The statistical significance of three or more experiments was determined by performing a two-tailed, unpaired Student's *t*-test for two comparisons.

To confirm that PEA3 was overexpressed in MDA-MB-231 cells, immunoprecipitation (IP) followed by Western blot analysis was performed to detect PEA3 protein (Figure 3A). The IP followed by Western blot analysis showed that endogenous PEA3 could not be detected in pcDNA3.1-transfected MDA-MB-231 cells. However, cells transfected with the *PEA3 *expression plasmid expressed PEA3 protein (Figure 3A). The ChIP analysis was performed on cells transfected with vector alone or with *PEA3 *expression plasmid (Figures 3B and 3C). Endogenous PEA3 was not enriched in either the *Notch-1 *or *Notch-4 *promoter regions in cells transfected with pcDNA3.1. This could be due to weak antibody avidity or, more likely, could be a result of PEA3 protein instability [61]. PEA3 enrichment was found in both *Notch-1 *and *Notch-4 *promoter regions within 1 kb of the start site when PEA3 was overexpressed compared to vector alone-transfected cells (Figures 3B and 3C). A PEA3 siRNA smart pool was used to confirm the specificity of PEA3 recruitment and the PEA3 antibody. Figure 4A shows that PEA3 siRNA significantly decreased the enrichment of PEA3 in the identified promoter regions of *Notch-1 *and *Notch-4 *compared to a control siRNA. The Western blot shown in Figure 4B confirms that the overexpressed PEA3 in MDA-MB-231 cells was almost completely knocked down using the PEA3 siRNAa smart pool.

**Figure 5:**
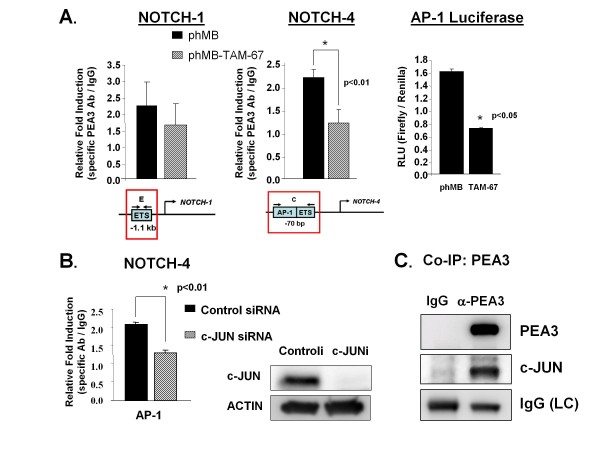
**The requirement of c-JUN for PEA3 enrichment on the Notch-4 promoter**. **(A) **MDA-MB-231 cells were cotransfected with pcDNA3.1-PEA3 and phMB vector alone or with phMB-TAM-67 expression plasmid. Lysates were subjected to chromatin immunoprecipitation using either PEA3-specific antibody or an isotype control IgG on the Notch-1 or Notch-4 promoter, respectively. PEA3 enrichment was measured by quantitative PCR and normalized to IgG control. In an independent experiment, cells were cotransfected with an AP-1 luciferase reporter, Renilla luciferase and phMB or phMB-TAM-67. Dual Firefly and Renilla luciferase assays were performed to measure AP-1 activity. Statistical comparisons were performed between phMB-vector and phMB-TAM-67 using a two-tailed, unpaired Student's *t*-test for two comparisons. **(B) **MDA-MB-231 cells were cotransfected with pcDNA3.1-PEA3 expression plasmid and control siRNA or c-JUN siRNA. Lysates were subjected either to chromatin immunoprecipitation and quantified as described above or to SDS-PAGE followed by Western blot analyses to detect c-JUN and actin (loading control). Statistical comparisons were performed between control siRNA and c-JUN siRNA using a two-tailed, unpaired Student's *t*-test for two comparisons. **(C) **PEA3 was immunoprecipitated from MDA-MB-231 lysates exogenously expressing PEA3 followed by Western blot analysis to detect PEA3 and c-JUN. The light chain of IgG was detected for loading purposes. The Western blots are representative of three independent experiments. Error bars represent standard deviations. The statistical significance of three or more experiments was determined by performing a two-tailed, unpaired Student's *t*-test for two comparisons.

To address whether PEA3 acts with AP-1 in *Notch-1 *and *Notch-4 *promoter regions, since it is known to work in accordance with c-Jun on several other genes [34,35,50], transfected lysates with either phMB-vector or phMB-TAM-67, a dominant-negative form of c-Jun missing the transactivation domain [62], were subjected to ChIP analysis. PEA3 enrichment on the *Notch-1 *promoter was not affected by TAM-67 (Figure 5A). However, PEA3 recruitment to an ETS site adjacent to an AP-1 site approximately 70 nt upstream from the start site of the *Notch-4 *promoter was significantly decreased by expression of TAM-67 (Figure 5B), indicating that the transactivation domain of c-Jun is required for PEA3 recruitment to the *Notch-4 *promoter. To confirm that TAM-67 reduces AP-1 transcriptional activity, an AP-1 luciferase reporter assay was performed in MDA-MB-231 cells transfected with either phMB vector alone or phMB-TAM-67. The results showed that the TAM-67-transfected cells contained more than 50% less AP-1 reporter activity than vector alone-transfected cells (Figure 5). To determine whether c-Jun specifically is required for PEA3 enrichment in the identified *Notch-4 *promoter region, transfected lysates with either control or c-Jun siRNA smart pool were subjected to ChIP analysis. In agreement with our previous findings, PEA3 enrichment in the *Notch-4 *promoter region was significantly decreased upon knockdown of c-Jun (Figure 5B), suggesting that c-Jun is required for PEA3 enrichment in the identified *Notch-4 *promoter region. We confirmed that c-Jun protein was knocked down by siRNA on the basis of Western blot analysis (Figure 5B). We also confirmed that c-Jun protein was in a complex with PEA3 in MDA-MB-231 nuclear extract by performing a co-IP assay (Figure 5C).

**Figure 6:**
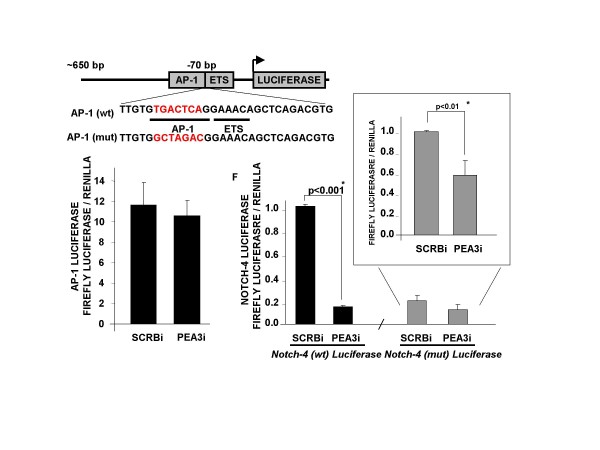
**PEA3 is required for Notch-4 reporter activity**. Schematic representation of the Notch-4 luciferase construct (-650 to +1 region) indicating the wild-type (wt) and mutated (mut) AP-1 consensus sites and the wild-type Ets site. MDA-MB-231 cells were cotransfected with AP-1 luciferase and either scrambled (SCRBia) or PEA3 siRNA (PEA3ia). Cells were cotransfected with either wild-type AP-1 or mutated AP-1 Notch-4 luciferase and either scrambled or PEA3 siRNAa. Firefly luciferase was measured normalized to Renilla luciferase as an internal transfection control. Means and standard deviations of three or more experiments were plotted. Statistical significance was determined by performing a two-tailed, unpaired Student's *t*-test.

To assess whether the results of the ChIP studies were not due to artificial interactions as a result of PEA3 overexpression, we performed a Notch-4 luciferase reporter assay using a minimal promoter region (-650 to +1) which contains the same AP-1/ETS region just -70 nt upstream from the start site. The wild-type construct contained an intact AP-1 and ETS consensus site at -70 nt. The mutant AP-1 construct contained an ablated AP-1 consensus site, but the adjacent ETS binding site was preserved [32]. Knockdown of endogenous PEA3 using siRNA significantly decreased wild-type AP-1-containing reporter activity (Figure 6, right graph) or mutant AP-1-containing reporter activity (Figure 6, inset) compared to the scrambled control. These results suggest that both PEA3 andAP-1 are critical for the regulation of the -70 nt region within the *Notch-4 *promoter in MBA-MD-231 cells. As a control study, we observed no significant difference in AP-1 luciferase reporter activity upon PEA3 knockdown, indicating that PEA3 was not mediating effects on the *Notch-4 *promoter indirectly through AP-1 regulation (Figure 6, left graph). Herein we provide the first evidence that both PEA3 and c-Jun are required to activate the *Notch-4 *promoter.

### Differential regulation of *Notch-4 *by AP-1 members

**Figure 7:**
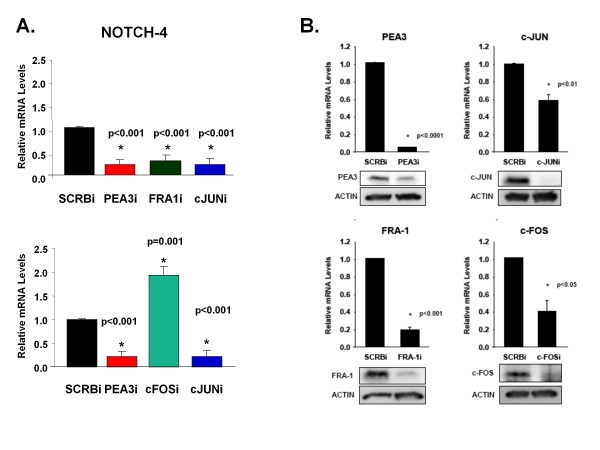
**Differential regulation of Notch-4 by AP-1 members**. **(A) **MDA-MB-231 cells were transfected with control, PEA3, Fra-1, c-JUN or c-FOS siRNA for 48 hours as described previously. Real-time PCR was performed to detect Notch-4 and HPRT transcripts. Transcript expression relative to control siRNA was calculated after normalization for HPRT transcripts. **(B) **Real-time PCR and Western blot analysis were performed to determine the efficacy of the specific siRNA. Means and standard deviations of three or more experiments were plotted. Statistical significance was determined by performing a two-tailed, unpaired Student's *t*-test for two comparisons and analysis of variance for multiple comparisons.

To determine which members of the Fos family of transcription factors are required for Notch-4 transcription, we performed real-time PCR to detect Notch-4 transcripts in response to knockdown of Fra-1 and c-FOS, which are the major forms of Fos in MDA-MB-231 cells. The results showed that Notch-4 transcripts were decreased 2.5-fold when Fra-1 was knocked down similarly to PEA3 or c-JUN knockdown (Figure 7A, upper graph). Conversely, c-FOS siRNA increased Notch-4 transcripts twofold compared to a scrambled control siRNA (Figure 7A, lower graph). Figure 7B demonstrates the efficacy of knockdown by PEA3, c-Jun, Fra-1 and c-FOS siRNA as measured by real-time PCR and Western blot analysis. These results, together with those of the previous ChIP studies, suggest that PEA3 regulates *Notch-4 *transcription by potentially interacting with c-Jun and Fra-1. The results also indicate that c-FOS is a potential transcriptional repressor of *Notch-4*.

### Dual inhibition of Notch and PEA3 inhibits cell proliferation and induces apoptosis

**Figure 8:**
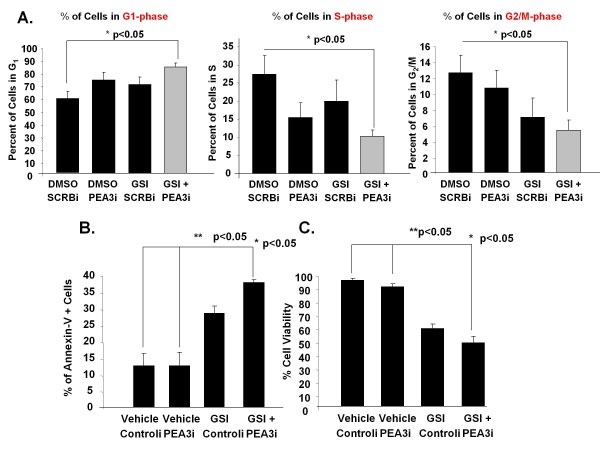
**Dual inhibition of Notch and PEA3 inhibits cell proliferation and induces apoptosis**. MDA-MB-231 cells were transfected with scrambled or PEA3 siRNA alone or were treated with vehicle (DMSO) or a γ-secretase inhibitor (MRK-003 GSI), or a combination thereof, for 48 hours. Cell cycle analysis **(A) **and annexin V staining **(B) **were performed by using flow cytometry. **(C) **Percentages of viable cells were measured by the trypan blue exclusion method using a standard light microscope (×10 original magnification). The mean percentage and standard deviation of cells in each experiment were plotted. Statistical significance was determined by performing analysis of variance for multiple comparisons. The error bars represent standard deviations of the mean for three independent experiments.

Notch is vital for proliferation and cell fate determination [63], whereas PEA3 is critical for cell migration and invasion [64,65]. Both aberrant and unregulated activities can lead to overall malignancy and poor survival [25,40]. To understand their biological significance and their effect in MDA-MB-231 triple-negative cells, we explored dual inhibition of PEA3 and Notch using the specific PEA3 siRNA smart pool and the preclinical MRK-003 GSI, respectively. MDA-MB-231 cells were transfected with scrambled control or PEA3 siRNA alone, treated with vehicle or MRK-003 GSI, or a combination of PEA3 siRNA plus MRK-003 GSI thereof for 48 hours. Independently, PEA3 knockdown or Notch inhibition had little effect on the cell cycle compared to control (Figure 8A). PEA3 knockdown or MRK-003 GSI treatment of MDA-MB-231 cells alone showed a modest but not significant increase in G_1 _phase arrest, a modest decrease in the S phase and little effect on the G_2_/M phase (Figure 8A). However, the combination of PEA3 knockdown and MRK-003 GSI treatment resulted in a significant increase in the G_1 _phase compared to control (*P *< 0.05) (Figure 8A). In the presence of both PEA3 knockdown and MRK-003 GSI treatment, there was a significant reduction in the S phase of the cell cycle compared to control (*P *< 0.05). Also, both PEA3 knockdown and MRK-003 GSI treatment resulted in a significant reduction in the G_2_/M phase of the cell cycle compared to control (*P *< 0.05) (Figure 8A). These results indicate that both PEA3 and Notch are critical for the proliferation of MDA-MB-231 cells.

We then asked whether dual inhibition using both PEA3 knockdown and Notch inhibition affect cell viability, potentially through increased apoptosis. Independently, PEA3 knockdown showed no change in apoptosis as measured by (1) annexin V staining (Figure 8B) or (2) cell viability using trypan blue exclusion (Figure 8C). MRK-003 GSI treatment alone increased apoptosis to 30% (Figure 8B) and decreased cell viability to 60% (Figure 8C). Importantly, the combination of PEA3 knockdown and MRK-003 GSI treatment significantly increased apoptosis to almost 40% as measured by positive staining of annexin V cells (Figure 8B) and diminished cell viability to 50% (Figure 8C). These results, taken together with the cell cycle data, indicate that both PEA3 and Notch activities are critical for cell proliferation and survival in MDA-MB-231 breast cancer cells.

### Dual inhibition of Notch and PEA3 inhibits anchorage-independent growth

**Figure 9:**
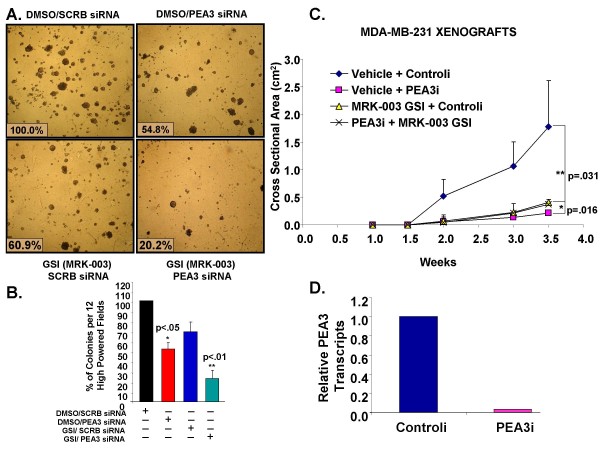
**Inhibition of Notch and PEA3 inhibits anchorage-dependent growth *in vitro *and tumor growth *in vivo***. **(A) **and **(B) **MDA-MB-231 cells were transfected with either scrambled or PEA3 siRNAa and treated with vehicle (DMSO) or γ-secretase inhibitor (MRK-003 GSI) independently or in combination for 48 hours. A colony formation assay was performed using methylcellulose, and the colonies were incubated for 14 days. **(A) **Colonies were photographed using a standard light microscope (×40 original magnification). The photomicrographs are representative of three independent experiments. **(B) **Twelve fields per well were counted, and the means plus or minus standard deviations of three independent experiments were plotted. Statistical significance was determined by performing analysis of variance for multiple comparisons. **(C) **Cells were injected into each of two mammary fat pads of female athymic nude mice which were number-tagged prior to surgery to monitor each tumor. The mice were randomized to vehicle or MRK-003 GSI, which was fed orally by gavage on a schedule of three days on, four days off. Tumor areas (length × width) were measured twice per week using vernier calipers for up to 3.5 weeks. The *y*-axis is cross-sectional in centimeters squared, which is calculated using the formula [(area × π)/4]. The *x*-axis is weeks of treatment. The graph shows the mean cross-section of the tumors plus or minus the standard deviations of 20 tumors/10 mice/group. Linear regression analysis was performed on individual tumors to calculate the slope of a line with a correlation coefficient >0.85. Statistical analysis was performed using a two-tailed, unpaired Student's *t*-test on the slopes of each line. *Denotes statistical differences between vehicle + PEA3i and vehicle + Controli. **Denotes statistical differences between MRK-003 GSI + Controli and vehicle + Controli. **(D) **MDA-MB-231 cells were transfected with control or PEA3 siRNAa for 24 hours. Real-time PCR was performed on MDA-MB-231 cells prior to injection to detect PEA3 transcripts.

To address whether dual inhibition of PEA3 and Notch activities affect anchorage-independent growth as an *in vitro *measure of tumorigenicity, we performed a colony formation assay with MDA-MB-231 cells that were transfected with either scrambled control or PEA3 siRNA and treated with vehicle or MRK-003 GSI independently or in combination. We observed a reduction of almost 55% in the number of colonies when PEA3 was knocked down or 61% when Notch was inhibited using a GSI in cells compared to vehicle + control siRNA control (Figures 9A and 9B). Furthermore, the number of colonies was reduced further to 20% upon PEA3 knockdown and GSI treatment compared to vehicle + control siRNA (Figures 9A and 9B).

### Tumor growth of MDA-MB-231 xenografts is dependent on PEA3 or Notch activity

The results so far have indicated that dual inhibition of PEA3 and Notch signaling inhibits both anchorage-dependent and anchorage-independent cell growth *in vitro *more effectively than either treatment alone. The results from the anchorage-independent assay suggest that transient knockdown of PEA3 using siRNA is sufficient to inhibit the formation of colonies for up to two weeks. On the basis of these results, we asked whether transient knockdown of PEA3 could inhibit tumor formation *in vivo *and whether treatment with MRK-003 GSI in combination could prevent tumor formation. We performed an orthotopic MDA-MB-231 tumor xenograft study in athymic mice. MDA-MB-231 cells were transfected with control or PEA3 siRNA *in vitro *(Figure 9D) and then injected into the mammary fat pads of female athymic mice. The mice were randomized to injection with vehicle control or MRK-003 GSI. The results showed that either PEA3 knockdown or GSI treatment significantly reduced tumor growth by almost similar rates compared to vehicle + scrambled control siRNA (Figure 9C).

We have shown for the first time, to our knowledge, that PEA3 is a novel activator of Notch-1 and Notch-4 transcription in different subtypes of breast cancer cells and could prove to be an important therapeutic target, possibly upstream of Notch-1 and/or Notch-4 signaling.

## Discussion

PEA3 was originally identified as a member of the ETS family of transcription factors. Since then, it has been observed that PEA3 is expressed during normal breast development, is quickly lost upon maturation and yet reemerges in metastatic breast cancers [33,54,61,66-68]. Similarly, Notch has been implicated in breast cancer, demonstrating elevated expression and activity in breast tumors [25,26]. Little is known about the factors that influence *Notch *gene expression and why its levels are elevated in breast cancer. Herein we provide the first evidence of transcriptional regulation of *Notch-1 *and *Notch-4 *by PEA3 in MDA-MB-231 and other breast cancer subtypes (Figures 1 through 3). We have demonstrated that *Notch-4 *gene regulation is dependent on AP-1 factors such as c-Jun, Fra-1, c-Fos and now PEA3, depending on cellular context (Figures 5 and 7). Interestingly, we provide evidence that c-Jun and Fra-1 are transcriptional activators of *Notch-4*, but that c-FOS could be a transcriptional repressor of *Notch-4 *(Figure 7). PEA3 regulates the *Notch-1 *promoter, but the additional factors that aid in that regulation are still being investigated. Further evidence reveals that PEA3 inhibition helps sensitize MDA-MB-231 cells to a GSI, showing promise in significantly reducing both anchorage-dependent and anchorage-independent growth as well as increasing apoptosis *in vitro *(Figures 8, 9A and 9B). Moreover, PEA3 expression or Notch activity is required for optimal growth of MDA-MB-231 tumors *in vivo *(Figure 9C). Thus, PEA3 emerges as a potential target, possibly upstream of Notch activity, for triple-negative cancer and possibly other breast cancer subtypes where PEA3 and/or Notch activities are critical for growth. This was evident in the preclinical model of our MDA-MB-231 xenograft study (Figure 9C). Either PEA3 knockdown or GSI treatment was adequate to significantly inhibit tumor growth *in vivo*. This result could suggest that targeting PEA3, a transcriptional activator of Notch-1 and Notch-4, hits multiple targets at once. For example, PEA3 is an activator of *MMPs *[36-39,53], *uPAR *[55,56], *COX-2 *[35] and now *Notch-1 *and *Notch-4*. PEA3 expression and Notch signaling could be critical for tumor formation and communication with the tumor microenvironment, which is not possible to recapitulate *in vitro *using single-cell suspensions. The severe side effects associated with GSI treatment, such as gastrointestinal toxicity [69,70], could possibly be avoided if inhibition of PEA3 is able to inhibit several growth- and metastasis-promoting signaling pathways.

Notch is a cellular fate determinant and can induce cell proliferation and/or differentiation, depending on the cellular environment [71]. PEA3 has been linked to the invasion, migration and aggressiveness of tumor cells [33,40,53,54,57,61,67,68,72,73]. The dual or individual inhibition of Notch by the GSI, and the inhibition of PEA3 by siRNA, acts by preventing two vital arms of cancer progression, namely, growth and possibly invasion, which we are currently investigating *in vitro *and *in vivo*. Emerging nanotechnology can be used as a means by which to direct siRNA therapies [74], and the advent of stapled interface peptides [75] that disrupt transcription factor complexes transforms the notion of specific targeting of the PEA3 transcription factor into a potential reality.

In addition, Harrison *et al*. [27] implicated Notch-4 in mammary tumor stem cell survival and self-renewal in a recent study in which they demonstrated that targeting Notch-4 specifically was more effective than a targeting a GSI in inhibiting the Notch pathway. In our studies, we found that Notch-4 gene transcription was more sensitive to PEA3 inhibition. Given this fact, the sensitivity that we obtained in our MDA-MD-231 system by the dual inhibition using a PEA3 siRNA in combination with MRK-003 GSI reduced viability and increased apoptosis may be explained by the notion that we may have targeted not only the proliferation and survival of bulk cancer cell populations but also possibly the cancer stem cell population.

Herein we have provided evidence of a novel therapeutic strategy to be exploited for the treatment of triple-negative breast cancer and potentially other breast cancer subtypes where PEA3 regulates Notch-1 and Notch-4. Enhanced sensitivity toward current GSIs or alternative strategies for future clinical trials by inhibition of PEA3 by nanoparticles, small-molecule inhibitors or future siRNA approaches may increase patient response to treatment and could reduce or eliminate recurrence if stem cell populations are eliminated. Inhibiting PEA3 may also allow for a larger therapeutic window for GSI treatment, enabling the reduction of pharmacological doses or possibly eliminating the need for the GSI if PEA3 is indeed upstream of Notch signaling, thus lowering resultant undesirable side effects such as gastrointestinal toxicity and possibly skin cancer.

## Conclusions

Taken together, the results from this study demonstrate for the first time that *Notch-1 *and *Notch-4 *are novel transcriptional targets of PEA3 in breast cancer cells. PEA3 emerges as a potential innovative target upstream from Notch activity for triple-negative cancer and possibly other breast cancer subtypes where PEA3 and/or Notch activities are critical for growth and aggressive phenotypes. The significance of targeting of PEA3 and/or Notch pathways allows a potentially novel therapeutic strategy for the treatment of breast cancers.

## Abbreviations

AP-1: activator protein 1; DMEM: Dulbecco's modified Eagle's medium; ERα: estrogen receptor α; GSI: γ-secretase inhibitor; IL: interleukin; MMP: matrix metalloprotease; NEC: extracellular notch; NFκB: nuclear factor κB; NIC: intracellular notch; NTM: transmembrane notch; PBS: phosphate-buffered saline; PCR: polymerase chain reaction; PEA3: polyomavirus enhancer activator 3; SCRBi: scrambled control siRNA; siRNA: small interfering RNA;

## Competing interests

AGC and CO declare that there is a patent pending on the identification of PEA3 as a biomarker for sensitivity to GSIs in breast tumors and possibly other solid tumors in conjunction with work conducted with the MRK-003 GSI developed by Merck Oncology International, Inc. We declare that there are no other financial conflicts of interest besides the patent application.

## Authors' contributions

AGC performed the majority of the experiments and wrote the manuscript. AR and KP injected MDA-MB-231 cells into mice, fed mice MRK-003 orally by gavage and assisted in tumor area measurements and statistical calculations. LM is a co-principal investigator on CO's US Department of Defense award and provided reagents and critical expertise. All authors read and approved the final manuscript.
